# Inoculation of *Triatoma Virus* (*Dicistroviridae: Cripavirus*) elicits a non-infective immune response in mice

**DOI:** 10.1186/1756-3305-6-66

**Published:** 2013-03-15

**Authors:** Jailson F B Querido, Jon Agirre, Gerardo A Marti, Diego M A Guérin, Marcelo Sousa Silva

**Affiliations:** 1 Centre for Malaria and Tropical Diseases - Instituto de Higiene e Medicina Tropical - Universidade Nova de Lisboa, Lisboa, Portugal; 2 Unidad de Biofísica (UBF, CSIC-UPV/EHU), Barrio Sarriena S/N, 48940, Leioa, Bizkaia, Spain; 3 C Fundación Biofísica Bizkaia, Barrio Sarriena S/N, Leioa, Bizkaia, 48940, Spain; 4 Centro de Estudios Parasitológicos y de Vectores (CEPAVE-CCT-La Plata-CONICET - UNLP) 2-584, La Plata, 1900, Argentina; 5 Departamento de Bioquímica y Biologia Molecular, Universidad del País Vasco (UPV/EHU), 48940, Leioa, Spain

**Keywords:** *Dicistroviridae*, *Triatoma virus*, Chagas disease, triatomines, *Trypanosoma cruzi*, Mice immune response

## Abstract

**Background:**

*Dicistroviridae* is a new family of small, non-enveloped, +ssRNA viruses pathogenic to both beneficial arthropods and insect pests. Little is known about the dicistrovirus replication mechanism or gene function, and any knowledge on these subjects comes mainly from comparisons with mammalian viruses from the *Picornaviridae* family. Due to its peculiar genome organization and characteristics of the *per os* viral transmission route, dicistroviruses make good candidates for use as biopesticides. *Triatoma virus* (TrV) is a pathogen of *Triatoma infestans* (*Hemiptera: Reduviidae*), one of the main vectors of the human trypanosomiasis disease called Chagas disease. TrV was postulated as a potential control agent against Chagas’ vectors. Although there is no evidence that TrV nor other dicistroviruses replicate in species outside the *Insecta* class, the innocuousness of these viruses in humans and animals needs to be ascertained.

**Methods:**

In this study, RT-PCR and ELISA were used to detect the infectivity of this virus in *Mus musculus* BALB/c mice.

**Results:**

In this study we have observed that there is no significant difference in the ratio IgG2a/IgG1 in sera from animals inoculated with TrV when compared with non-inoculated animals or mice inoculated only with non-infective TrV protein capsids.

**Conclusions:**

We conclude that, under our experimental conditions, TrV is unable to replicate in mice. This study constitutes the first test to evaluate the infectivity of a dicistrovirus in a vertebrate animal model.

## Background

*Dicistroviridae* is a recently established family of small, non-enveloped viruses, containing a +ssRNA genome, and is classified under the order *Picornavirales*. This family contains 14 members classified within two genera, *Cripavirus* (type species *Cricket paralysis virus*, CrPV), and *Aparavirus* (type species *Acute bee paralysis virus*, ACP) [1,2]. A third genus has been recently proposed, *Triatovirus* (type species *Triatoma virus*, TrV) [3]. All dicistroviruses are pathogenic to arthropods, although primarily to insects, representing significant threats to the health of beneficial arthropods such as *acute bee paralysis virus*, *Black queen cell virus*, *Kashmir bee virus*, which infects honeybees [4-6], and *Taura syndrome virus*, which is pathogenic to shrimps [7]. Two dicistrovirus members infect the model organism *Drosophila melanogaster*, *Drosophila C virus* (DCV) and CrPV [8,9], and eight of them are pathogenic to insect pests, as it is the case with CrPV, which infects field crickets and the *Olive Fruit Fly* as well [7]. The aforementioned CrPV is quite ubiquitous and replicates in many other insect species spanning five orders [10]. Although dicistroviruses are believed to be arthropod-specific, antibodies against CrPV have been detected in sera from a pig, horse, and cattle [11]. Similarly, high levels of anti-TrV antibodies were detected in chickens used to feed a colony of TrV-infected insects, although the authors of this work concluded that the chickens were apparently refractory to the infection with TrV [12].

Compared to other families of picornaviruses, little is known about dicistrovirus infection, replication mechanism or gene function. Most information about cellular infection and the replication cycle of dicistroviruses comes from studies on DCV, and by comparison with mammalian picornaviruses [1]. Many members of the *Dicistroviridae* family are considered novel candidates to be used as biopesticides [1,13-17].

TrV and *Solenopsis invicta virus*-1 are the two lone members of the *Dicistroviridae* family that infect insects of medical importance [1]. In fact, TrV is a natural enemy of *Triatoma infestans* (*Hemiptera: Reduviidae*), one of the main vectors of the trypanosomiasis disease called Chagas disease, a severe human illness most prevalent in almost all Latin American countries [18,19]. To date, TrV is the only entomopathogenic virus found in triatomines [20]. The viral particles are spherical, with a diameter of 30 nm [21], and consist of a non-enveloped capsid that encloses a 9010 nt long viral genome [22]. The capsid contains four structural proteins VP1 (29.7 kDa), VP2 (28.4 kDa), VP3 (31.8 kDa) and a minor one VP0 (37.3 kDa). In addition to these four structural proteins, this virus has a low molecular weight protein of 5.5 kDa that appears to be detached from the capsid, lying at the particle interior and presumably in contact with the genome [3,20,23]. TrV can also infect natural populations of *T. sordida* and several experimental populations of triatomines as well [24]. Viral replication takes place in the midgut epithelium cells of triatomines, causing many deleterious sublethal effects in *T. infestans* and *T. patagonica* colonies, such as reduction of the longevity, increase of the developmental time, a decrease in both fecundity and fertility of eggs, and even a cumulative mortality higher than 97% [16,25-28].

As pointed out previously, the infectivity of TrV in vertebrates remains unclear. Therefore, in this work we studied the infectivity of TrV in mice (*Mus musculus* - BALB/c mice). Our results indicate that inoculations of both infective and non-infective TrV particles elicit the same immune response, and that this insect virus is unable to replicate in this animal model. Based on the bicistronic character of dicistrovirus genomes, it was speculated that these pathogens may not infect vertebrates, and this report constitutes important experimental evidence to support that hypothesis.

## Methods

### *Triatoma virus* purification

Virus purification was performed according to [3]. Dry feces obtained from infected insects were collected on Whatman filter papers and excised from them using a standard surgery scalpel until two g of material was collected. A buffer composed of 10 mM NaCl, 1 mM MgCl_2_ and 200 mM citric acid at pH 6 (Lysis buffer) was added at a ratio of 100 mL per gram of dry material. PMSF (N 98.5%, purchased from Sigma) thawed at −20°C in isopropanol (Merck, GR for analysis) was added up to a final concentration of 1 mM. The mixture was homogenized for 5 min using a vortex mixer and then sonicated using a Sanyo MSE Soniprep 150 operating at 10 s ON/10 s OFF for 20 pulses. After centrifugation, the resulting pellet was resuspended in NMT buffer and then loaded on top of a 35 mL continuous 5%–30% sucrose gradient. The gradients were cooled in ice to avoid particle diffusion within them. The individual optical density of each aliquot was then measured at both 260 nm and 280 nm. After plotting the absorbance profile, selected aliquots were loaded onto 12.5% polyacrylamide SDS-PAGE gels to check the purity of the samples. Following gradient fractionation, equivalent samples were pooled together and dialyzed overnight against 2 L of NMT buffer.

### Infection with *Triatoma virus* (TrV)

Eight groups of female *Mus musculus* BALB/c mice (3 mice per group) between 5 and 8 weeks of age obtained from Instituto de Higiene e Medicina Tropical, Lisbon, Portugal, were used in the experimental infection with TrV. Groups 1-5 were inoculated by intraperitoneal injection. Group 1 is the control group, which was inoculated only with PBS. Group 2 was inoculated with 3.0 μg of empty TrV particles (capsids without genome, therefore non-infective) [3]. Group 3 was inoculated with 3.0 μg of TrV, group 4 and group 5 with 0.3 μg and 0.03 μg of TrV respectively. Group 6 served as a negative control of *per os* inoculation, therefore it was inoculated only with 100 μl of PBS delivered *per os* intraesophageally by using a 1 ml tuberculin syringe and Gavage needle. Group 7 was inoculated with 3.0 μg of empty TrV particles in 100 μl of PBS, delivered *per os* intraesophageally by using a 1 ml tuberculin syringe and Gavage needle. Group 8 was inoculated with 3.0 μg of TrV in 100 μl of PBS, delivered *per os* intraesophageally by using a 1 ml tuberculin syringe and Gavage needle. After inoculation blood and feces samples were collected at different times (3, 7, 30 and 45 days). Forty-five days after inoculation, the animals were sacrificed.

All experiments on animals were conducted with prior approval from the Animal Welfare of General Management of Veterinarians (DGV - Portugal). All manipulations of mice satisfied the requirements of the Regulations of Experimental Animal from the Ethics Committee of IHMT/UNL-Portugal.

### Searching for TrV in blood and feces samples

Blood samples from each mouse were collected into individual micro tubes containing 5 μl of Heparin-Sodium (B. Braun, USA). The RNA was extracted according to the product manual supplied with the RNA isolation Kits, (Bioline, UK). RNA concentration was determined by measuring absorbance at 260 nm (Abs_260_) in a spectrophotometer (NanoDrop 1000, Thermo Scientific).

Feces samples were collected into micro tubes. For each group of mice we pooled the samples from all mice of this group. 1,2 mg of fecal samples resuspended in 200 μl PBS were homogenized and centrifuged (5,000 rpm) for 10-15 minutes. 50 μl of supernatant was then homogenized in TRIZOL reagent (Genbiotech, Argentina), and viral RNA-TrV (vRNA) was purified according to the manufacturer’s instructions. As a positive control of RNA extraction, we carried out the same procedure simultaneously with feces from triatomines infected with TrV.

Purified vRNA (0.724 μg) was used as a template for the positive control in the RT-PCR reaction. For the other RT-PCR, we used 5 μl of the samples resulting from the purification of the vRNA from blood and feces samples from the mice used in the infectivity experiment. The cDNA synthesis was performed according to the cDNA Synthesis Kit protocol (Bioline, UK). 5 μl of the extraction product was used according to the OneStep RT-PCR protocol (QIAGEN; USA). TrV positive PCR reactions have a 832 bp product according to the protocol established in our previous work [26]. As a positive control of RT-PCR and RNA extraction from blood samples, we performed a PCR with two primer pairs from β-actin gene: β-actin sense: 5´ TGGAATCCTGTGGCATCCATGAAAC 3´ and β-actin anti-sense: 3´ TAAAACGCAGCTCAGTAACAGTCCG 5´, with an expected product of 348 bp [29]. PCR products were visualized on 1.5% agarose gels for TrV and 2% agarose gel for β-actin, stained with ethidium bromide, and their sizes were determined by comparison against DNA markers, HyperLadder I (Bioline, UK) and HyperLadder IV (Bioline, UK) respectively.

### Production of mouse polyclonal antibodies to anti-TrV

The serum used as a positive control in ELISA reactions were produced in female *Mus musculus* BALB/c mice between 5 and 8 weeks of age. The mice were inoculated (subcutaneous injection) with 100 μg of empty TrV particles mixed with 100 μl of Freund’s complete adjuvant (Sigma-Aldrich, USA). Fifty days after the first inoculation, the animals were inoculated with 100 μg of empty TrV particles mixed with 100 μl of Freund’s incomplete adjuvant (Sigma-Aldrich, USA). Ten days after the second inoculation, the animals were sacrificed and hyperimmune sera was obtained.

### Detection of anti-TrV IgGs antibodies by ELISA

Naturally empty TrV particles were selected as antigens for their protein-only composition. These particles contain almost the same proteins as full particles, but at least 30% of its composition was integrated by 7 additional polypeptides that were determined to be misprocessed products from the cleavage of the structural protein precursor P1 [3]. Nevertheless, since both full and empty particle proteins share the same origin (P1), their antigenic properties remain essentially the same.

Three different concentrations of total proteins (50, 100 and 150 ng/well) from the empty TrV particles were used to optimize the amount of antigen used in this reaction. We chose 100 ng/well because with this value the amount of antigen is no longer the limiting reagent of the reaction. Total protein extract from empty TrV particles (in carbonate buffer pH 8.5) was adsorbed overnight at 4°C onto 96-well micro-plates (Nunc, Denmark) for use in indirect enzyme-linked immunosorbent assays (ELISA), using sera from inoculated mice. Subsequently, horseradish peroxidase, (HRP)-conjugated rat anti-mouse IgG (1:4000, Sigma- Aldrich, USA), rat anti-mouse IgG1 (500 ng/ml, AbD serotec, UK) or rat anti-mouse IgG2a (250 ng/ml, AbD serotec, UK) were used.

## Results

### Absence of clinical infection signals

Mice inoculated with TrV by intraperitoneal and *per os* route did not show any behavioral alteration or clinical signals of viral infection (e.g. leg paralysis, change in food intake rate, weight loss, decrease in motility and death), when compared with mice inoculated with empty TrV particles or saline solution (control groups), this is contrary to what happens in triatomines, where the insects inoculated with the virus show leg paralysis, delayed development and death [16,22].

### Absence of TrV in blood and feces samples

**Figure 1:**
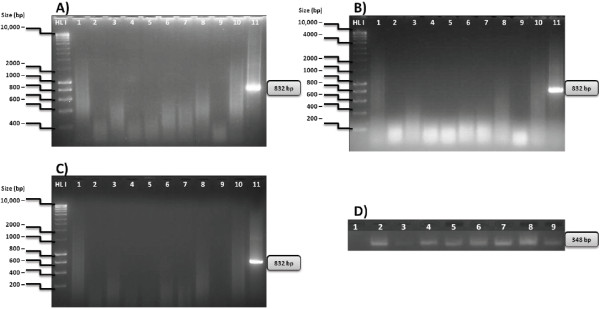
**A), B) and C) RT-PCR products with TrV primers on 1.5% agarose gel stained with ethidium bromide, at zero, three and forty-five days after inoculation respectively.** HL I: 200 bp to 10000 bp; Lane 1: negative control of RNA extraction; 2: IP with PBS; 3: IP with 3 μg empty TrV particles; 4: IP with 3 μg TrV; 5: IP with 0.3 μg TrV; 6: IP with 0,03 μg TrV; 7: Per os with PBS; 8: Per os with 3 μg empty TrV particles; 9: Per os with 3 μg TrV; 10: Negative control of RT-PCR, 11: 12.5 ng of pDNA (TrV). **D**) RT-PCR products with β-actin primers on 2% agarose gel stained with ethidium bromide forty-five days after inoculation. HL IV: 100 bp to 1000 bp; Lane 1: negative control of RNA extraction; 2: IP with PBS; 3: IP with 3 μg empty TrV particles; 4: IP with 3 μg TrV; 5: IP with 0,3 μg TrV; 6: IP with 0.03 μg TrV; 7: Per os with PBS; 8: Per os with 3 μg empty TrV particles; 9: Per os with 3 μg TrV.

We analyzed all blood and feces samples from each group of animals at 0, 3 and 45 days after inoculation. No RT-PCR products corresponding to TrV were detected from blood or feces samples of any of the groups of mice inoculated with TrV or mice inoculated with empty TrV particles (Figure 1). All blood samples showed PCR products corresponding to β-actin gene, with expected size (348 bp) (Figure 1-D).

### Detection anti-TrV IgGs antibodies by ELISA

We performed ELISA to detect antibodies against TrV in sera extracted from inoculated mice. Total protein extract from empty TrV particles was used to detect the presence of anti-TrV antibodies in sera from non-inoculated and inoculated BALB/c mice with TrV. We produced polyclonal antibodies to anti-TrV in mice in order to establish basal and positive signals in the ELISA. The cut-off value was defined using sera from non-inoculated mice, mean OD (optical density) + 3SD (standard deviation) at 490 nm. We chose this weighting (3SD), because all sera from non-inoculated mice had OD values less than the *cut-off* value.

**Figure 2:**
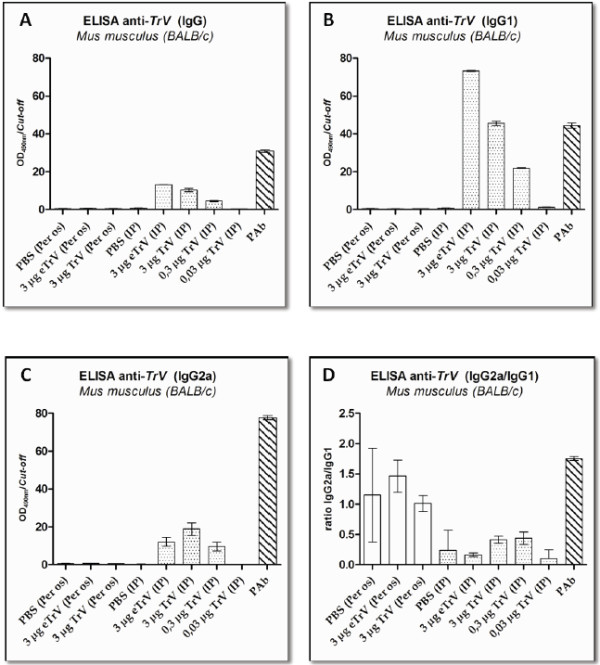
**A) Levels of total IgG anti-TrV antibodies, B) Levels of total IgG1 anti-TrV antibodies, C) Levels of total IgG2a anti-TrV antibodies elicited in mice BALB/c inoculated with empty TrV particles (eTrV) and RNA-full TrV capsids (TrV); D) ratio IgG2a/IgG1 antibodies.** Serum samples were taken on days 45 after injection. Each column represents the mean with standard deviation (SD) from the ratio of optical density (OD_490 nm_) and cut-off values (three mice per group). Cut-off value was defined as: mean + 3SD. Abbreviations: IP: intraperitoneal route; PAb: Polyclonal antibodies.

Forty-five days after intraperitoneal inoculations, all inoculated mice showed high levels of IgG anti-TrV antibodies when compared with sera from non-inoculated mice (Figure 2) or mice inoculated by *per os* route. Sera reactivity was directly proportional to the amount of antigen used in inoculation. However, there was no significant difference (F = 0.0064; p-value > 0.05) between the reactivity of sera from mice inoculated with empty TrV particles to that of mice inoculated with the same amount of RNA-full TrV capsid (Additional file 1). All mice inoculated by the *per os* route presented levels of anti-TrV antibodies similar to mice inoculated only with PBS (Figure 2), indicating that *per os* inoculation with TrV produces little or no IgG antibody response in mice.

To define which subclasses of IgG anti-TrV were detected in sera from inoculated mice and in order to determine if TrV can replicate in mice, we measured the titers of IgG1 and IgG2a antibodies specific to TrV (Figure 2-B and C). The results show that there is no significant difference in the IgG2a/IgG1 ratio between mice inoculated with TrV, mice inoculated with empty TrV particles or non-inoculated mice (Figure 2-D).

## Discussion

RT-PCR has been used to detect a variety of RNA viruses in different kinds of samples [9,30]. In this study, a RT-PCR assay has been developed to detect TrV from blood and feces samples from mice inoculated with TrV. Two specific pairs of primers for TrV were used and the 832 bp products expected for the TrV-specific assay were produced only when TrV vRNA was present, indicating that this assay is highly specific for TrV.

In this PCR assay, PCR products were detected from samples with low concentrations of plasmid DNA (6.2 pg) encoding specific TrV sequences or TrV vRNA (2.5 ng) (data not shown). Therefore, if TrV were able to replicate in mice, their presence would be detected by RT-PCR assay from blood and feces samples of inoculated mice. However, we were not able to detect any PCR products from blood or fecal samples of mice inoculated with TrV. The absence of PCR products from these samples may indicate that TrV is unable to replicate in mice, this contrasts to what happens in triatomines, where this virus causes a high mortality rate, reduces fecundity and delays the development of infected insects [16,25].

It has been postulated that the immune response triggered by viruses mainly activates the Th1 subset of T helper cells and enhances the production of INF-γ, which is the reason many systemic viral infections in mice result in preferential increases in virus-specific IgG2a antibodies in serum [31,32]. This preferential class switch is dependent on viral replication and is attributed in part to virus-induced production of IFN-γ [31]. In this study we observed that there is no significant difference in the ratio IgG2a/IgG1 in sera from animals inoculated with TrV when compared with non-inoculated animals or mice inoculated only with empty TrV particles. The results reported in this work support the idea that TrV is unable to replicate in vertebrates.

## Conclusions

The results of the ELISA for anti-TrV together with the results of a TrV vRNA search by RT-PCR in blood and feces samples from mice inoculated with TrV indicate that this virus is not infective in mice, at least under the conditions explored in this study (infected by *per os* and intraperitoneal inoculations). The results reported in this work support the idea that TrV is a virus that only infects invertebrates.

To date, this study constitutes an important approach to evaluate the response of one vertebrate animal model to the experimental infection with a dicistrovirus. Future studies may lead to a better understanding of the TrV host range, and hopefully may also characterize the effect observed in humans and other vertebrates that are most likely to be exposed to dicistroviruses [10,22]. Certainly, to improve the current knowledge on TrV, and in general, to gain information on the life cycle of any member of the *Dicistroviridae* family, will greatly help in establishing a proof of principle for employing dicistroviruses as biological agents to control insect pests.

## Competing interests

The authors declare that they have no competing interests.

## Authors’ contributions

Conceived and designed the experiments: MSS, DMAG, GAM. Performed the experiments: JBQ, JA, MSS. Analysed the data: JBQ, GAM, MMAG, MSS. Wrote the paper: JBQ, DMAG, MSS. All authors have read and approved the final manuscript.

## Supplementary Material

Additional file 1 **Statistical analysis (ANOVA, with Microsoft Excel®) between the two groups of mice inoculated with the same concentration of RNA-full TrV capsids and empty TrV particles respectively (3 μg).** Considering that the data is normally distributed, this test helped to identify differences between mice inoculated with TrV and mice inoculated with the same amount of empty TrV particles. The null hypothesis (H_0_) assumes that the means are statistically the same (H_0_: μ_1_ = μ_2_), and the alternate hypothesis (H_A_) assumes that the means are statistically different (H_A_: μ_1_ ≠ μ_2_), at 95% confidence. Since the F statistic is smaller than the critical value, we fail to reject the null hypothesis. Sum of squares (SS); Degrees of freedom (df); Mean square (MS). Click here for file
