# Supplementary material for: Inoculation of Triatoma Virus (Dicistroviridae: Cripavirus) elicits a non-infective immune response in mice
Source: Parasit Vectors. 2013 Mar 15;6:66. doi: 10.1186/1756-3305-6-66 (PMC3605389; doi:10.1186/1756-3305-6-66)
Supplement: Additional file 1 — Statistical analysis (ANOVA, with Microsoft Excel®) between the two groups of mice inoculated with the same concentration of RNA-full TrV capsids and empty TrV particles respectively (3 μg). Considering that the data is normally distributed, this test helped to identify differences between mice inoculated with TrV and mice inoculated with the same amount of empty TrV particles. The null hypothesis (H0) assumes that the means are statistically the same (H0: μ1 = μ2), and the alternate hypothesis (HA) assumes that the means are statistically different (HA: μ1 ≠ μ2), at 95% confidence. Since the F statistic is smaller than the critical value, we fail to reject the null hypothesis. Sum of squares (SS); Degrees of freedom (df); Mean square (MS). [file 1756-3305-6-66-S1.pdf]

Source of Variation	*SS*	*df*	*MS*	*F*	*P-value*	*F crit*	*Significant*
Between Groups	0.0009	1	0.0009	0.0064	0.9403	7.7086	NO
Within Groups	0.5904	4	0.1476				
Total	0.5913	5					
